# Expression of secretory leukocyte protease inhibitor detected by immunohistochemistry correlating with prognosis and metastasis in colorectal cancer

**DOI:** 10.1186/1477-7819-12-369

**Published:** 2014-12-02

**Authors:** Guiying Liu, Jingyan Yang, Yulei Zhao, Zhijing Wang, Baoheng Xing, Liang Wang, Dongliang Shi

**Affiliations:** Department of Delivery Room, Cangzhou Central Hospital, 16 Xinhua West Road, Yunhe District, Cangzhou City, Hebei Province 061001 People’s Republic of China; Department of Anesthesiology, Cangzhou Central Hospital, 16 Xinhua West Road, Yunhe District, Cangzhou City, Hebei Province 061001 People’s Republic of China; Department of Hematology, Cangzhou Central Hospital, 16 Xinhua West Road, Yunhe District, Cangzhou City, Hebei Province 061001 People’s Republic of China; Department of Cerebral Surgery, Cangzhou Central Hospital, 16 Xinhua West Road, Yunhe District, Cangzhou City, Hebei Province 061001 People’s Republic of China; Department of Maternity, Cangzhou Central Hospital, 16 Xinhua West Road, Yunhe District, Cangzhou City, Hebei Province 061001 People’s Republic of China; Institute of Radiation Medicine, Chinese Academic of Medical Sciences & Peking Union Medical College, 238 Baidi Road, Nankai District, Tianjin, 300192 People’s Republic of China; Department of Oncology, Cangzhou Central Hospital, 16 Xinhua West Road, Yunhe District, Cangzhou City, Hebei Province 061001 People’s Republic of China

**Keywords:** Colorectal cancer, Immunohistochemistry, Metastasis, Prognosis, Secretory leukocyte protease inhibitor

## Abstract

**Background:**

The potential of secretory leukocyte protease inhibitor (SLPI) as a biomarker for colorectal cancer was studied. A prospective, randomized, controlled, clinical trial was conducted in 2013 and 2014 to confirm whether the expression of SLPI correlates with prognosis and metastasis in colorectal cancer patients.

**Methods:**

Immunohistochemistry was used to detect SLPI expression in colorectal cancer. The expression of SLPI was scored by two pathologists independently. Statistical analysis of the data was performed using a Χ^2^ test to investigate the influence of SLPI on the pathologic characteristics of colorectal cancer.

**Results:**

Compared with normal tissue, SLPI was overexpressed in colorectal cancer tissue. Overexpression of SLPI correlated with different grades (moderate or good differentiation: 2.7% low expression versus 97.3% high expression, low differentiation: 41.7% low expression versus 58.3% high expression), TNM stage (I or II: 4.2% low expression versus 95.8% high expression; III or IV: 19.7% low expression versus 80.3% high expression), lymphatic metastasis (18.6% low expression versus 81.4% high expression) and distal metastasis (86.5% low expression versus 13.5% high expression), but not with patient age or sex (*P* = 0.613, *P* = 0.871).

**Conclusions:**

Upregulated SLPI correlates with aggressive pathologic characteristics of colorectal cancer; SLPI could be used as an indicator of progression and metastasis in patients with colorectal cancer.

## Background

Colorectal cancer is one of the most common malignancies worldwide and is a major cause of cancer-related deaths [1]. Survival rates of patients with colorectal cancer have increased in the past few years, possibly as a result of earlier diagnosis and improved treatment regimens; nonetheless, approximately 30% to 50% of patients who undergo curative resection subsequently experience local tumor recurrence or metastasis [2, 3].

The secretory leukocyte protease inhibitor (SLPI) is an 11.7-kDa, nonglycosylated, single-chain protein that is produced by different cell types, including lung epithelial cells, secretory cells of the salivary glands, and various host inflammatory and immune cells, such as macrophages, neutrophils, and B lymphocytes [4–7]. However, its potential as biomarker for colorectal cancer has not been studied.

This study was a prospective, randomized, controlled, clinical trial conducted in 2013 and 2014 to confirm whether SLPI expression correlates with prognosis and metastasis in colorectal cancer patients.

## Methods

### Tumor tissues

A uniform cohort of 296 patients (162 men and 134 women) with colorectal cancer (6 with stage I, 112 with stage II, 132 with stage III and 46 with stage IV cancer) diagnosed between January 2013 and March 2014 were selected. All patients provided their consent for participation in the study, which was approved by the local ethics committee. The age of the patients ranged from 24 to 87 years (mean: 53.3 years). Table 1 shows patients’ detailed information. Two cores of normal mucosa and two cores of tumor tissue for each patient were paraffin-embedded in ordered microarrays. Tumor microarrays were sectioned in preparation for immunohistochemical staining. Staining intensity was scored by two pathologists separately. A score of 1 was used to signify low intensity, a score of 2 signified intermediate intensity, and a score 3 signified high intensity. A score of 0 to 2 indicates low SLPI expression and a score of 3 indicates high SLPI expression [8].

**Table 1:** **Relationship between expression of SLPI and its clinic pathological characteristic in colorectal cancer**

	Patients	SLPI expression (%)		Χ^2^	***P***
		High	Low		
**Sex**				0.013	0.871
**Male**	162	76 (46.9)	86 (53.1)		
**Female**	134	65 (48.5)	69 (51.5)		
**Age (years)**				0.241	0.613
**>65**	104	49 (47.1)	55 (52.9)		
**<65**	192	101 (52.6)	91 (47.4)		
**Pathologic grade**				38.143	<0.05
**Good or moderate**	260	253 (97.3)	7 (2.7)		
**Low**	36	21 (58.3)	15 (41.7)		
**TNM grade**				19.241	<0.05
**I–II**	118	113 (95.8)	5 (4.2)		
**III–IV**	178	143 (80.3)	35 (19.7)		
**Lymphatic metastasis**				25.174	<0.05
**Positive**	129	105 (81.4)	24 (18.6)		
**Negative**	167	164 (98.2)	3 (1.8)		
**Distal metastasis**				31.247	<0.05
**Positive**	37	5 (13.5)	32 (86.5)		
**Negative**	259	232 (89.6)	27 (10.4)		

### Tissue preparation and staining

Specimens were fixed in 10% paraformaldehyde. Paraffin-embedded tissue microarrays were incubated at 60Â°C prior to de-waxing and rehydration. Antigen retrieval was achieved by placing sections in 10 Î¼M citric acid (pH 6) and microwaving for 15 minutes. Endogenous peroxidases were quenched in 15 ml hydrogen peroxide and 185 ml of water. Samples were washed again with PBS prior to treatment with Starting Block (Thermo Scientific, Rockford, IL) for 10 minutes. Tissues were incubated in mouse anti-human SLPI monoclonal antibody (1:50; Santa Cruz, American). The primary antibodies were diluted in PBT (10Ã— PBS, 10% BSA, 10% Triton X-100 in double distilled H_2_O) at 4Â°C overnight. Samples were washed with PBS on the following day, incubated in secondary antibody (1:1000), washed and then treated using a 3,3'-diaminobenzidine (DAB) peroxidase staining kit (Shanghai Biotechnology Company, NO132, Xuhui District, Shanghai City, China) as per the manufacturer’s protocol. For detection, a DAB peroxidase detection kit was used and color development was monitored using an optical microscope. The development process was terminated by removing DAB and rinsing the sections with ddH_2_O for 1 min prior to counterstaining with hematoxylin.

### Statistical analysis

Data are representative of three independent experiments. SPSS20.0 software was used for statistical analysis. Statistical analysis of the data were performed using the Χ^2^ test. The differences were considered significant at *P* < 0.05.

## Results

**Figure 1:**
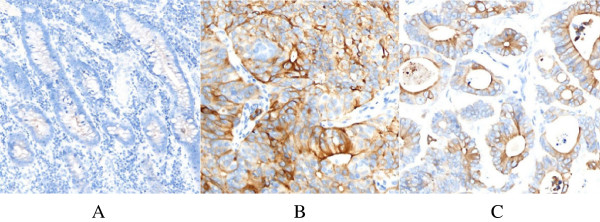
**Expression of SLPI in different tissues. (A)** Normal colon tissue; **(B)** poorly differentiated colon cancer; **(C)** well differentiated colon cancer.

In Figure 1, we can see negative expression of SLPI in normal colon tissue (Figure 1A) and positive expression of SLPI in poorly and well differentiated colon cancer (Figure 1B,C).

As shown in Table 1, the expression of SLPI was not correlated with age or sex (*P* = 0.613, *P* = 0.871). The positive expression of SLPI was positively correlated with the degree of tumor differentiation (*P* < 0.05). There is significant correlation between SLPI and the TNM stage of the tumor. The rate of positive expression in patients with stage III or IV cancer was significantly higher than that in stage I or II (*P* < 0.05). Moreover, the expression of SLPI in patients with distant metastasis is higher than that without metastases (*P* < 0.05).

## Discussion

The secretory leukocyte protease inhibitor gene is located on chromosome 20q12-13.1 in human beings and on chromosome 2H in mice, with a similar exon-intron configuration [8]. Although physiologically unique and designated as a protease inhibitor, *SLPI* has the structural characteristics of a WFDC domain. Chromosome 20q13 was recently recognized as the WFDC locus, containing genes encoding 14 WFDC-type protease inhibitors [9]. Secretory leukocyte protease inhibitor, which has antimicrobial and anti-protease functions, belongs to the whey acidic protein four-disulfide core family of proteins, and is also produced in cancer tissues, but its role in cancer is not well understood [10].

Bouchard *et al.* [11] reported that SLPI expression is highly upregulated in pancreatic, papillary thyroid, uterine cervix, endometrial, and ovarian cancer; by contrast, SLPI is underexpressed in nasopharyngeal carcinoma, bladder tumors, and some breast carcinomas, although overexpression of this protein correlates with more invasive forms of breast carcinoma. Devoogdt *et al.* [12] reported that several factors, such as inflammatory cytokines and steroid hormones, affect *SLPI* gene expression. Hoskins *et al.* [13] reported that SLPI stimulates ovarian cancer invasion, modulated in part by its serine protease inhibitory activity, attenuating MMP-9 release.

This study investigated the potential clinical utility of SLPI to serve as a prognostic and metastasis-predictive biomarker in patients with colorectal cancer. We performed independent validation experiments using a large cohort of samples from 296 patients with colorectal cancer. These data provide strong evidence that SLPI is overexpressed in colorectal cancer tissue. Overexpression of SLPI is correlated with tumor grade and TNM stage, but not with patient age or sex. Our data are of particular interest because they highlight that overexpression of SLPI provides a predictor of lymphatic metastasis and distant metastasis.

## Conclusions

The high expression level of SLPI detected by immunohistochemistry in colorectal cancer showed that it correlated with poorly differentiated colorectal cancer with a TNM stage of III or IV, lymphatic metastasis and distal metastasis. Collectively, these results indicate that evaluation of SLPI expression in patients with colorectal cancer presents a clinically promising biomarker that can facilitate disease risk assessment and severity in patients with colorectal cancer. It could be used as an indicator for progression and metastasis of colorectal cancer. However, further studies should be undertaken to verify whether SLPI can become a new indicator for colorectal cancer recurrence and survival of patients.

## Abbreviations

BSA: bovine serum albumin
DAB: 3,3'-diaminobenzidine
PBS: phosphate buffered saline
PBT: 10Ã— PBS, 10% BSA, 10% Triton X-100 in double distilled H_2_O
SLPI: secretory leukocyte protease inhibitor.
